# Phototunable Biomemory Based on Light‐Mediated Charge Trap

**DOI:** 10.1002/advs.201800714

**Published:** 2018-06-25

**Authors:** Ziyu Lv, Yan Wang, Zhonghui Chen, Long Sun, Junjie Wang, Meng Chen, Zhenting Xu, Qiufan Liao, Li Zhou, Xiaoli Chen, Jieni Li, Kui Zhou, Ye Zhou, Yu‐Jia Zeng, Su‐Ting Han, Vellaisamy A. L. Roy

**Affiliations:** ^1^ College of Electronic Science and Technology Shenzhen University Shenzhen 518060 P. R. China; ^2^ Department of Materials Science and Engineering and State Key Laboratory of Millimeter Waves City University of Hong Kong Tat Chee Avenue, Kowloon Hong Kong SAR 999077 China; ^3^ State Key Laboratory of Advanced Technology for Materials Synthesis and Processing Wuhan University of Technology 122 Luoshi Road Wuhan 430070 P. R. China; ^4^ State Key Laboratory of Transducer Technology Shanghai Institute of Microsystem and Information Technology Chinese Academy of Sciences Shanghai 200050 China; ^5^ Institute for Advanced Study Shenzhen University Shenzhen 518060 P. R. China; ^6^ College of Optoelectronic Engineering Shenzhen University Shenzhen 518060 P. R. China

**Keywords:** carbon dots, charge trapping, Kelvin probe force microscopy, resistive switching, silk

## Abstract

Phototunable biomaterial‐based resistive memory devices and understanding of their underlying switching mechanisms may pave a way toward new paradigm of smart and green electronics. Here, resistive switching behavior of photonic biomemory based on a novel structure of metal anode/carbon dots (CDs)‐silk protein/indium tin oxide is systematically investigated, with Al, Au, and Ag anodes as case studies. The charge trapping/detrapping and metal filaments formation/rupture are observed by in situ Kelvin probe force microscopy investigations and scanning electron microscopy and energy‐dispersive spectroscopy microanalysis, which demonstrates that the resistive switching behavior of Al, Au anode‐based device are related to the space‐charge‐limited‐conduction, while electrochemical metallization is the main mechanism for resistive transitions of Ag anode‐based devices. Incorporation of CDs with light‐adjustable charge trapping capacity is found to be responsible for phototunable resistive switching properties of CDs‐based resistive random access memory by performing the ultraviolet light illumination studies on as‐fabricated devices. The synergistic effect of photovoltaics and photogating can effectively enhance the internal electrical field to reduce the switching voltage. This demonstration provides a practical route for next‐generation biocompatible electronics.

Prominent improvements in communication and information technologies generate the exponential growth of data‐storage device requirement due to the fundamental and critical functions of memory‐storage units for contemporary computing system.^1^, ^2^, ^3^, ^4^, ^5^, ^6^, ^7^, ^8^, ^9^, ^10^ Analysts predict that total memory demand (i.e., 3 × 10^24^ bits) will exceed commercial semiconductors supply in 2040.^11^, ^12^ At the same time, an urgent ecological issue occurs due to vast amounts of unrecyclable and environmental e‐waste are produced and abandoned annually.^13^, ^14^ Pressures arising from the tremendous require amount of data‐storage devices, coupled with a green strategy to solve global environmental problems, have inspired the development of more creative and environmentally friendly solutions.^15^, ^16^ In this respect, state‐of‐the‐art, bioinspired resistive random access memory (RRAM) based on biomaterials has been considered as effective strategy to solve the urgent ecological issue stemmed from increased amount of annually abandoned unrecyclable e‐waste.^17^, ^18^, ^19^ Biodegradable natural materials with diverse electrical and mechanical properties can be employed as data‐storage layer in memories to promise an alternative approach to replace conventional inorganic semiconductors. In addition, biomaterials allow a biocompatible interface between electronic devices and biological worlds, thus broadening corresponding biotechnological and medicinal applications, such as implantable chips, artificial neurons, and electronic skin.^20^, ^21^, ^22^, ^23^, ^24^ Various types of biomaterials have been used as active units for fabrication of data‐storage devices, such as protein, polysaccharide, nucleic acid, and virus.^25^, ^26^, ^27^, ^28^, ^29^, ^30^ Due to versatile properties, such as biodegradability, biocompatibility, bioresorbability, optical transparency, and light weight, proteins, the most readily accessible biomolecules can be used as an attractive building blocks for the development of RRAM devices and endow these electronic memories with excellent performance and environmental benignity.^31^, ^32^ They have been both served as an active switching component of a passive substrate to construct two‐terminal RRAM devices with impressive ON/OFF ratios, ultralow density, low operating voltage, good degradability, and tunable‐resistive switching behavior.^33^, ^34^, ^35^, ^36^, ^37^, ^38^, ^39^

In another aspect, to endow programming bit precision with biomemories will offer significant advantages for the further application of bio‐RRAM, such as artificial synapse and neurons.^40^, ^41^, ^42^ As a noninvasive stimulus, photoprogramming allows the possibility of remote control memory devices with nearly arbitrary spatial and temporal precision, suggesting prominently enhanced operation speed and a localized control capability of different electronic components separately.^43^, ^44^, ^45^ Furthermore, a phototunable resistive switching platform may endow a novel understanding of memory operations.^46^

Nevertheless, to the best of our knowledge, the protein‐based phototunable biomemories and systematical study of its underlying mechanism have not been reported yet. As an ancient biomaterials, silk is one kind of proteins with distinguished mechanical and optical properties,^38^ while the biocompatible carbon dots (CDs) have emerged as promising materials for optoelectronic applications because of their small size, attractive optical properties, and low preparation cost.^47^, ^48^, ^49^ In this article, we report photoactive biomemories based on CDs‐silk composite in which various metal anodes including Al, Au, and Ag were used to systematically study the resistive switching behavior. Discrepancy of the memory characteristics is observed in Al, Au‐based, and Ag‐based biomemory. Incorporation of CDs with light‐adjustable charge trapping capacity was found to be responsible for with phototunable‐resistive switching properties of CDs‐based RRAM by performing the ultraviolet (UV) light illumination studies on as‐fabricated devices. The obvious decreasing trend of switching voltage and on current was observed under UV irradiation. Finally, in situ analyzing charge carrier trapping behaviors in Al/CDs‐silk/indium tin oxide (ITO) and Au/CDs‐silk/ITO devices via Kelvin probe force microscopy (KPFM), and conductive filament formation/rupture in Ag/CDs‐silk/ITO device via scanning electron microscopy and energy‐dispersive spectroscopy (SEM/EDS) were further investigated to explore the detailed photoresponsive switching mechanism.

First, total reflectance Fourier transform and circular dichroism spectra were utilized to investigate the silk spatial conformation due to a great impact of secondary structure of silk protein in its resistive switching behavior.^50^ **Figure** **1**a shows the characteristic absorption peaks of amide I (1700–1600 cm^−1^) and amide II (1600–1500 cm^−1^) region of silk protein thin film. A strong absorption band at 1653 cm^−1^ indicates that the silk matrix adopts α‐helix conformation. In addition, the circular dichroism spectrum further confirmed α‐helix conformation in the silk (Figure 1b). A positive peak at 192 nm and two negative peaks at 208 and 222 nm indicate the predominance of α‐helix structure. As shown in Figure 1c,d, transmission electron microscope (TEM) and atomic force microscope (AFM) images of pristine CDs display spherical morphology with a uniform diameter of ≈20 nm. The hexagonal lattice in the fast Fourier transform pattern image of the CDs (inset of Figure 1c) indicates that CDs possess crystalline hexagonal structures. The UV–vis absorption peaks of CDs aqueous solution at 243 and 342 nm are respectively attributed to π–π* transition and n–π* transition (Figure 1e). Under excitation at wavelengths from 330 to 390 nm, CDs exhibit the strongest peak at 452 nm at λ_ex_ = 360 nm (Figure 1f). The inset images display the optical images of CDs solution that display yellow color under illumination of visible light and blue fluorescent color under irradiance of UV light which further verifies the strong photoluminescence of CDs. The topographic AFM images of silk‐CDs composite film are displayed in Figure 1g. CDs‐silk film fabricated with optimal doping ratio and annealing temperature exhibits uniform distribution of CDs in a smooth surface with 0.445 nm average roughness (*R*_a_) and 0.574 nm root mean square roughness (*R*_rms_).

**Figure 1:**
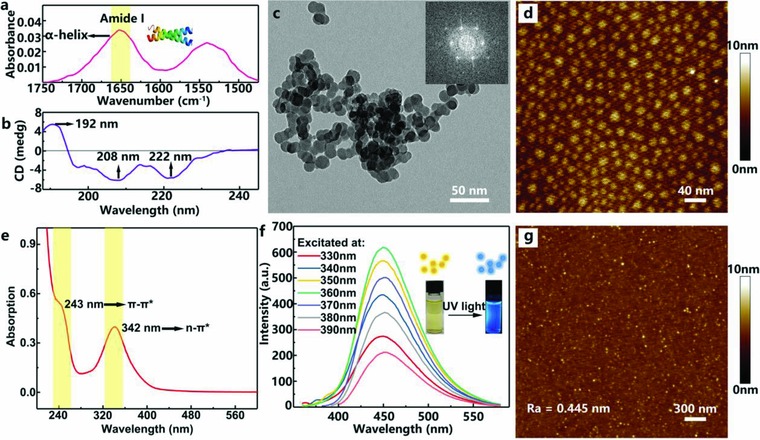
Materials characteristics. a) Fourier transform infrared spectroscopy‐attenuated total reflectance (FTIR‐ATR) spectrum of amide I and amide II region of silk protein thin film, the yellow ribbon indicates the characteristic vibration peak of silk protein: δ_C = O_. b) Circular dichroism spectrum of silk protein in aqueous solution (concentration: 0.05 mg mL^−1^). c) TEM images of pristine CDs. The inset is the fast Fourier transform pattern of CDs. Scale bar: 50 nm. d) AFM topographic images of pristine CDs on a mica substrate. Scale bar: 40 nm. e) UV–vis spectrum of CDs aqueous solution (concentration: 0.05 mg mL^−1^). f) Fluorescence spectra of CDs aqueous solution (concentration: 0.003 mg mL^−1^) at different excitation wavelengths. Inset: Photographs and schematic illustration of CDs solution before and after UV light irradiation (λ: 365 nm). g) AFM topographic images of CDs‐silk composite film on a mica substrate. Scale bar: 300 nm.

**Figure** **2**a shows the 3D schematic illustration of CDs‐silk memory devices. A CDs‐silk film is spin coated on glass substrate with predeposited 200 nm ITO electrode. After thermal annealing of the film, different parallel top electrodes including Al, Au, and Ag are thermal‐evaporated using a shadow mask separately. As depicted in Figure 2b, the cross‐sectional SEM image of device with Al top electrode demonstrated that the CDs‐silk layer is sandwiched between the bottom ITO and top Al electrode. The blend ratio and concentration influences on electrical characteristics and switching effects were carried out (see Figures S2 and S3 in the Supporting Information for more details). Optimal ratio of CDs to silk with the CDs concentration of 0.5 mg mL^−1^ and silk concentration of 10 mg mL^−1^ was employed to fabricate the active layer of bio‐RRAM. We first explored the electrical characteristics of the bio‐RRAM devices with different top electrodes. The typical current–voltage (*I–V*) curve based on Al top electrode was first measured in sweeping mode with a 1 mA compliance current. As shown in Figure 2c, beginning at 0 V bias without UV illumination, the Al/CDs‐silk/ITO device starts in a high‐resistance state (HRS) until reaching 3.1 V, where the current suddenly increased up to six orders of magnitude, indicating an electrical transition from the HRS to the low‐resistance state (LRS). During the subsequent positive and negative bias sweeps, the device stays in the ON state, indicating a write‐once read‐many‐times (WORM) memory behavior. By adjusting the compliance current from 1 to 100 mA, similar WORM memory behavior with non‐negligible fluctuation of SET voltage was observed. As displayed in Figure 2d, quite similar WORM characteristics and impact of compliance current on operating voltage are observed in the Au/CDs‐silk/ITO devices. Nevertheless, compared with Al/CDs‐silk/ITO devices, much higher ON/OFF current are found in CDs‐silk based devices with top Au electrodes. This may result from a lower potential barrier height (0.26 eV) between CDs‐silk layer and Au electrode than that between active layer and Al electrode (0.54 eV), as indicated by the potential difference between Al and Au electrode in contrast with CDs‐silk layer through a KPFM measurement (Figure 2f). It is worth noting that Ag/CDs‐silk/ITO device exhibits a reversible bipolar‐resistive switching property by an ultralow SET and RESET voltage which is in discrepancy with the Au‐ and Al‐based biomemories. During the positive SET sweep at *I*cc of 1 mA, the device maintains HRS until reaching ≈0.5 V, where the current suddenly increases from 1.55 × 10^−10^ to 7.37 × 10^−7^ A, indicative of a switching from the HRS to LRS1 (Figure 2e). As the bias increase to ≈1 V, the device experiences another resistance state decreased by three orders of magnitude, indicating two ON state are obtained (LRS1 and LRS2) in the device. The two‐step transition from HRS to LRS may be understood as follows: A bicomponent blend is used as the active layer, which consists of inorganic carbon dots doping and silk protein matrix, to fabricate the biomemory devices. The heterogeneous film prepared by the bicomponent blend may cause discrepant Ag ion migration capacity, which then leads the formation of Ag conductive biochannels with different threshold voltage in CDs‐silk insulating layer. During reverse sweep RESET process, the OFF state can be recovered at the voltage of ‐0.9 V and then maintained during the rest of the RESET sweep. We find that the device cannot be reset back if the higher *I*cc (>1 mA) is employed during the SET operation which is originated from the extremely robust conductive path formed at high compliance currents. In addition, the dependence of device ON and OFF current on cell area was investigated (see Figure S4 in the Supporting Information). The OFF current is size dependent and proportional to the cell area, which indicates the same current passing through the whole cell. However, the ON current is size independent, suggesting the ON state is mainly attributed to localized conductive channel.

**Figure 2:**
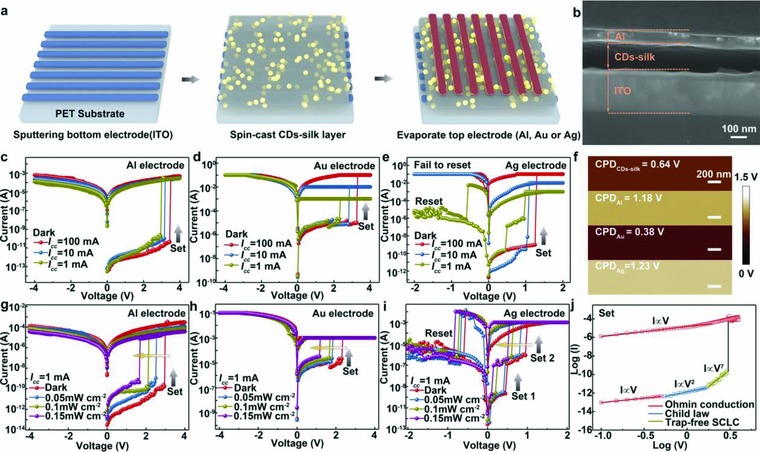
Device fabrication, structure, and *I*–*V* characteristics. a) Schematic illustration depicting the fabrication process of the flexible CDs‐silk memory devices. b) Cross‐sectional SEM image of the device structure. Scale bars: 100 nm. c–e) *I*–*V* characteristics of CDs‐silk memories with c) Al, d) Au, and e) Ag top electrode at different positive compliance current (dark, sweeping rate: 50 mV). f) Contact potential difference images from KPFM measurement of CDs‐silk film, Al surface, Au surface, and Ag surface. g–i) *I*–*V* characteristics of CDs memories with g) Al, h) Au, and i) Ag top electrode, exposed to UV light (λ = 365 nm) with intensity from 0 to 0.15 mW cm^−2^ (sweeping rate: 50 mV). j) Experimental data and fitted lines of the *I*–*V* characteristics of the Al/CDs‐silk/ITO device at SET process without light irradiation (compliance current: 1 mA).

Studies were then performed on UV light irradiation effects on the memory characteristics. As displayed in Figure 2g, switching voltage of Al/CDs‐silk/ITO device steadily decreases from 3.1, 2.5, 2.0, to 1.6 V when illumination intensity increased from 0, 0.05, 0.10, to 0.15 mW cm^−2^ during SET operation. The ON current is found to exhibit similar decreasing trend from 2.09 × 10^−4^ to 9.82 × 10^−6^ A with UV irradiance increased from 0 to 0.15 mW cm^−2^. This photoinduced decreasing in operating voltage and ON current of RRAM may offer advantages in the development of low power data‐storage devices. Figure 2h,i depicts quite similar UV illumination‐induced negative shift of SET voltage in both Au/CDs‐silk/ITO and Ag/CDs‐silk/ITO devices.

To evaluate the reproducibility, we investigated the distribution of switching voltages and ON/OFF current (*I*_on_ and *I*_off_) by measuring 100 randomly selected memory cells of Al/CDs‐silk/ITO device. **Figure** **3**a exhibits the statistical distribution of switching voltage with and without UV light irradiation. As indicated by the Gaussian fitting results, the average values of switching voltage without and with light irradiation were 3.1 ± 0.8 and 1.8 ± 0.6 V, respectively. Furthermore, histogram of current in Figure 3b,c manifests a smaller value and a narrower distribution of *I*_on_ with light irradiation than that of *I*_on_ under dark condition. UV light irradiation effect on SET voltage and high reproducibility of Au/CDs‐silk/ITO and Ag/CDs‐silk/ITO device were also proved by analyzing the distribution of switching voltages in Figures S5 and S6 in the Supporting Information. The stability of ON and OFF current without and with UV light irradiation is also tested with eclipse of time (Figure 3d–f). Compared with other reported biomemory systems, our CDs‐silk devices exhibit excellent reliability under both dark condition and UV irradiation, and no visible degradation of ON/OFF ratio is observed after 10^6^ s. The endurance of Ag/CDs‐silk/ITO device is demonstrated in Figure S7 in the Supporting Information. No obvious fluctuation is observed for the Ag‐based device after 100 SET and RESET sweep cycles.

**Figure 3:**
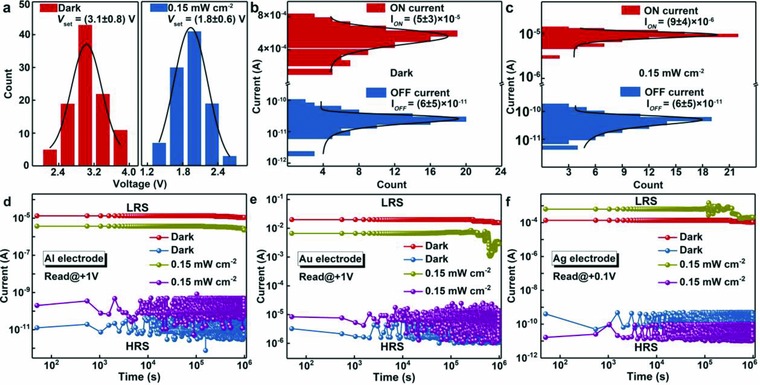
Device statistics and endurance. a) Distribution of SET voltages without (left) and with (right) UV light treatment (100 sample devices for both statistics histograms). The black lines are Gaussian fits to the distributions. Histogram of the ON and OFF current b) without and c) with light irradiation. The black lines are Gaussian fits to the histogram. d–f) The stability ON and OFF current of CDs‐silk memories with d) Al, e) Au, and f) Ag top electrode. The devices were measured with constant application read voltage at 300 K.

To understand the effect of light illumination on switching voltage, the switching mechanism of the Au and Al based bio‐RRAM memories should be clarified first. Conceivable‐resistive switching mechanisms in RRAM system consist of the formation of metal filament, the charge trapping/detrapping process, redox reaction of the insulating layer, or a combined mechanism.^51^, ^52^, ^53^, ^54^ First, we investigated the temperature dependence of the ON and OFF current of Al/CDs‐silk/ITO device (see Figure S8 in the Supporting Information). In both LRS and HRS, the current increases with the increased temperature, suggesting a semiconductive or insulating conductive behavior of CDs‐silk layer which exclude the possibility of switching stemmed from the formation of conductive metallic filament in CDs‐silk layer. This is in consistent with the poor Al/Au ionic migration capacity and the subsequent difficulty of formation Al/Au filament. Second, electrochemical analysis was conducted utilizing cyclic voltammetry to explore the redox properties of the CDs‐silk blend. Nevertheless, the cyclic voltammogram of the CDs‐silk film exhibits no visible oxidation/reduction peaks (see Figure S9 in the Supporting Information) so that no redox reaction takes place in the RRAM system can be confirmed. The experimental and fitted data for the positive voltage sweep were investigated in a log–log scale (Figure 2j). A nonlinear *I–V* curve containing four distinct regions with different linear slopes was observed, implying different conduction mechanism before and after resistive switching. The fitting data suggest that the carrier transport behavior follows the clear signature of space‐charge‐limited conduction space‐charge‐limited conduction (SCLC) mechanism.^55^ Initially, the slope of the fitting result is 0.97 at low bias, clearly suggesting Ohmic conduction mechanism. In this region, the density of injected charge carriers is less than that of the generated free carrier density. Then, with increasing the forward bias, the exponential of the fitted data is equal to 2, suggesting that the conduction is dominated by SCLC transport and obeys Child's law. Subsequently, a steep current increase behavior is observed, indicating a transformation from a trap‐infilled SCLC to a trap‐filled SCLC. For this region, the charge trap sites in CDs‐silk film are gradually filled by injected charge carriers. With increasing forward bias, the injected carriers gradually exceed the equilibrium concentration and the trap sites of the CDs‐silk layer are filled by the superabundant charge carriers, thus leading to the formation of charge carriers at the electrode interface. Hence, the SCLC‐dominated charge carrier transport process can be verified. Thus, the charge trapping/detrapping of CDs‐silk film is in charge of the resistive switching of Au‐ and Al‐based RRAM devices. The hopping conduction model could be utilized to illuminate the resistive switching mechanism. In HRS, the CDs‐silk layer shows an insulating property and contains some trap sites as a result of protein structural disorder, inhomogenous particle surface sites of CDs, and interface of CDs‐silk/electrode. With the treatment of applied voltage, the injected charge carriers will first fill the trap sites. Subsequently, at the switching voltage increases, these trap sites with encapsulated charge carriers will develop a conductive path in the initial insulating layer. On the other hand, considering that CDs can serve as excellent electron acceptors or donators under light irradiation, the charge trapping capacity of CDs‐silk may change after light irradiation, which affects the trapping/detrapping behavior in SET process and subsequently induces different switching voltage. This hypothesis needs subsequent investigation, which we will discuss in the following paragraph.

Microscaled carrier trapping and retain behavior inside the active layer were further performed by AFM electrical technique, which consists of a charge carrier injection process and a surface potential measurement (**Figure** **4**a). First, in a contact mode, a 1 µm × 1 µm square area was scanned with platinum‐/iridium‐coated conductive tip biased at negative or positive voltage to perform 2D carrier injection under dark or UV light illumination. At ‐6 V tip bias, negative electrons from a more negative point (AFM tip) flow are trapped in a more positive active layer, thus decreasing the surface potential of scanning area. Keep other parameters unchanged, hole injection is achieved by scanning another 1 µm × 1 µm square area with +6 V tip bias. Then, AFM is in situ switched into a KPFM mode with a 10 µm × 10 µm scanning area to monitor the spatial and temporal evolution of surface potential, thus evaluating carrier trapping and retain capacity of the active layer. Figure 4b displays the contact potential difference images between the conductive tip and CDs‐silk layer as a function of time after positively and negatively programming operation. Electron injection area displays decreased potential of 0.09 V and hole injection area shows increased potential of 0.03 V at the initial state (0 min), implying that CDs‐silk layer possesses a stronger trapping ability of electrons than that of holes. Figure 4c shows the line profiles of KPFM images in Figure 4b along the blue lines. With eclipse of time, initially trapped electrons exhibit 36% relaxation, while the initially trapped holes show 89% loss after 180 min. The better maintenance of electrons indicates that electrons have been trapped in the deeper level of active layer than holes. To evaluate the contribution of CDs in phototunable memory characteristic of CDs‐silk‐based biomemory, the charge‐trapping behavior of pristine silk layer and CDs‐silk composite layer under light illumination was investigated. Surface potential images of pristine silk, silk‐CDs with and without light illumination are depicted in Figure 4d,e. After doping CDs into silk film, the potential value of the uninjected area decreased from 1.30 to 0.62 V, and the potential change induced by electrons injection increased from 0.03 to 0.09 V, implying that the CDs play the vital roles in CDs‐silk medium. It is worth noting that the induced potential change of electron‐injected area increases from 0.09 to 0.15 V, while the value of hole injected areas displays negligible variation in silk‐CDs film after UV light illumination, suggesting that electron trapping capacity of the silk‐CDs film can be dramatically enhanced by the UV light illumination. Figure 4h,i depicts UV illumination enhance electron trapping capacity of the silk‐CDs medium.

**Figure 4:**
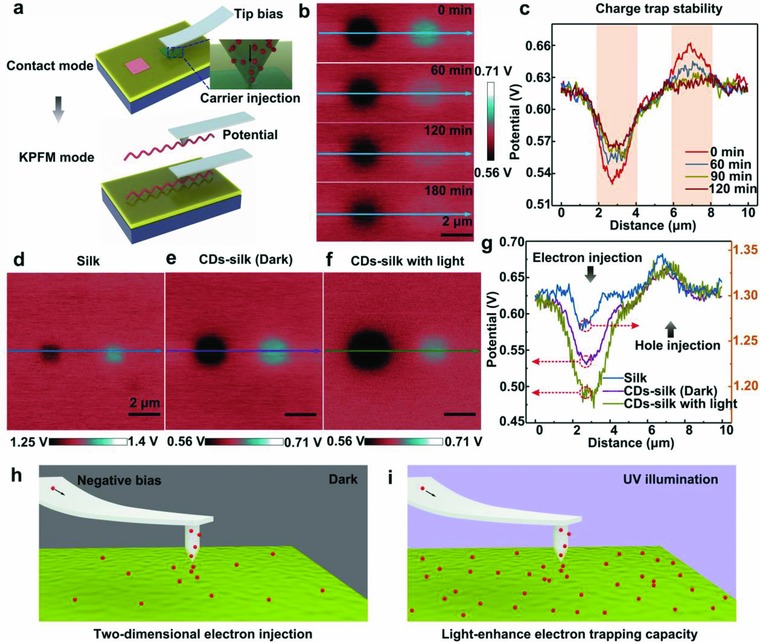
Charge trapping capability of CDs‐silk film detected by KPFM. a) Schematic representation of experimental setup for 2D injection of electrons or holes into CDs‐silk film in a contact mode, as well as subsequent surface potential measurement in a KPFM mode. A Pt/Ir‐coated conductive tip was used. (Charge injection area: 1 µm; tip bias: ‐6 V or +6 V; charge injections into CDs‐silk film without or with light were conducted by the same method; UV light intensity: 0.15 mW cm^−2^.) Surface potential images of b) CDs‐silk layer and c) selected cross section as a function of time (0, 60, 120, and 180 min) after charge injection process. Scale bars: 2 µm. Surface potential images of d) silk, e) CDs‐silk without or f) with light irradiation and g) the selected cross sections. Schematic illustration of the process of 2D electron injection to CDs‐silk film h) without and i) with UV illumination using a conductive AFM tip at negative bias.

To clarify the resistive switching mechanism in Ag anode‐based RRAM, the planar structure of Ag/CDs‐silk/Ag device with a 20 µm gap width which enabled a direct observation of conductive Ag filament growth in the CDs‐silk layer via microscopic techniques was fabricated. **Figure** **5**a displays the 3D illustration of as‐fabricated planar device and a typical *I–V* curve for the planar Ag/CDs‐silk/Ag device. During the subjection to the positive voltage scan (0 V →4 V), the device switches to LRS at 3.1 V, followed by an abrupt jump to the preset 1 mA compliance current. During the negative bias sweep, the device is switched off when the reverse voltage reaches –1.1 V. It is worth to note that the switching voltage of laterally structured device is much higher than that of vertically structured device since the strength of electrical field is negatively correlated to the thickness of CDs‐silk layer. Figure 5b shows the corresponding SEM images of the planar device focusing on the silk‐CDs film between the Ag electrode before and after application of positive bias. Evidently, the formation of a metallic filament in the CDs‐silk film between Ag electrodes can be detected after the SET operation. The EDS spectra collected from on regions 1–4 confirmed Ag element in the filaments and indicate the migration of Ag into silk‐CDs film from Ag electrode, which have been interpreted as electrochemical reactions at active anode (Figure 5c). In addition, the temperature dependence of the ON current of Ag/CDs‐silk/ITO device was also investigated. Figure 5d depicts the data retention characteristics of silk‐CDs‐based biomemory with respect to the temperature. A metallic behavior in which the LRS current decreased with the increased temperature is observed which is highly correlated to Ag conductive filament mechanism.

**Figure 5:**
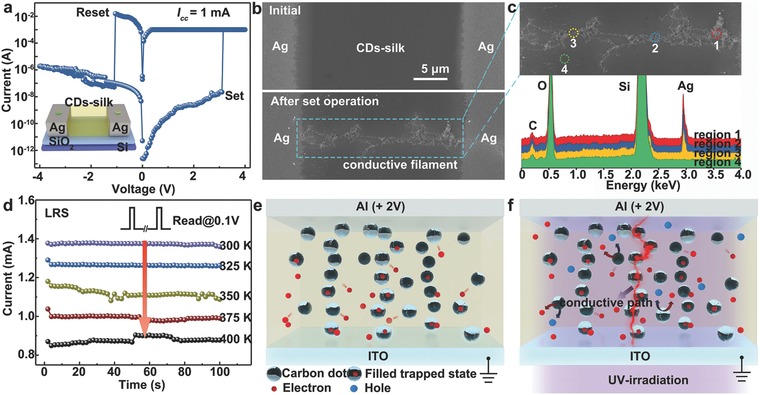
The working principle of CDs‐silk‐based memory device with Ag electrode and the proposed resistive switching mechanism in CDs‐silk‐based memory. a) The SET and RESET operation of a planar Ag/CDs‐silk/Ag device (compliance current: 1 mA, sweeping rate: 50 mV). The inset displays 3D illustration of as‐fabricated planar device. b) SEM images of the planar device before and after SET operation, scale bars: 5 µm. c) An enlarged SEM image of the conductive filament and EDS spectra in different regions. d) The temperature dependence of LRS currents of a vertical Ag/CDs‐silk/ITO device. The devices were measured with constant application of 0.1 V read voltage at 300–400 K. e,f) By application, a positive voltage sweep, the injected charge carrier begin to fill the trapping centers in the CDs‐silk layer. However, the bias (+ 2 V) is not enough to induce a conduction path. In contrast, the charge trapping capacity can be significantly enhanced via photogating effect induced by UV‐irradiation treatment, thus promoting the formation of a conductive path, leading to an abrupt change of device conductivity.

Based on the aforementioned analysis and observations, a possible‐resistive switching mechanism of CDs‐silk bio‐RRAM device can be visualized as below: for Al/CDs‐silk/ITO and Au/CDs‐silk/ITO device with anode materials exhibiting a higher first ionization potential, the injection and migration of metal cations fails since the oxidized form of the top electrode cannot be reduced back to the metal (Al) or the top electrode is not readily oxidized (Au).^56^ In addition, there are some trap sites in the CDs‐silk medium owing to inhomogenous particle surface sites of CDs, protein structural as well as the interface of CDs‐silk/electrode. Resistive switching is mainly originated from the charge trapping/detrapping inside the active layer. During a positive SET operation, the injected charges from electrode, particularly electron from ITO, will first fill the charge traps in the CD‐silk layer. Then, at a certain voltage, the charge traps with charge carrier trapped in them will form a stable conductive path, indicative of a switching from HRS to LRS. While the conductive path was too stable to be broken during a negative RESET operation since the electrons trapped in deeper level of CDs is hard to be released. This hypothesis also explains the WORM characteristics observed in the Au‐ and Al‐based devices. In another aspect, the formation/rupture of conductive Ag filament is the plausible mechanism for the resistive switching of Ag‐anode‐based device. Under forward positive bias, anodic dissolution of Ag occurs according to the reaction Ag→Ag^+^ + e^−^. The Ag^+^ migrates across the silk‐CDs film under the high electrical field which can be further reduced and electrocrystallized on the surface of cathode according to the reaction Ag^+^ + e^−^ →Ag. Eventually, the electrocrystallization process induces the formation of the Ag filaments growing preferentially in the direction of anode and a resistive switching from HRS to LRS. After the RESET operation, the electrochemical dissolution of Ag filament leads the LRS switching back to HRS.

Two distinct mechanisms are attributed to the phototunable memory behavior of bio‐RRAM. First, photovoltaic effect in which the electron–hole pair can be created in CDs through absorbing photons with higher energy compared with the band gap (Figure 5e,f). The excitons are divided in the interface between CDs and silk by an electric field. The photogenerated electrons are arrested by CDs and the interface is acting as a local gate. For Au/silk‐CDs/ITO and Al/ silk‐CDs/ITO devices, the photogating effect stemmed from trapped photogenerated electrons can strongly reduce the *V*set of device since the traps in the silk‐CDs film have been partially filled up at the initial state under UV illumination. While for Ag/silk‐CDs/ITO device, larger amount of Ag^+^ is repelled toward the cathode under enhanced electrical field. The formation process of Ag filament is accelerated by photogating effect which induces the decreasing *V*set of Ag/silk‐CDs/ITO under UV illumination as well.

In summary, we have proposed and demonstrated photonic bio‐RRAM based on biocompatible silk protein‐CDs composite. The Al‐, Au‐based biomemories exhibit WORM characteristics, while the reversible bipolar‐resistive switching is observed in Ag‐based device. Charge trapping/detrapping and conductive filament formation/rupture are attributed to resistive switching effect of the photonic memory devices based on inactive anode (Al or Au) and active anode (Ag), respectively, which have been verified by in situ KPFM nanotechnology and SEM/EDS microanalysis. The SET voltage and ON current are effectively tuned by performing the UV light illumination on as‐fabricated devices. The excellent biodegradability and biocompatibility of CDs‐silk switching materials enable a biocompatible interface between electronic devices and biological worlds, thus promoting the development of phototunable bioelectronic memory devices for potential applications in future implantable electronic devices, biosensors, and wearable systems.

## Conflict of Interest

The authors declare no conflict of interest.

## Supporting information

Supplementary
