# Supplementary material for: Phototunable Biomemory Based on Light‐Mediated Charge Trap
Source: Adv Sci (Weinh). 2018 Jun 25;5(9):1800714. doi: 10.1002/advs.201800714 (PMC6145401; doi:10.1002/advs.201800714)
Supplement: Supplementary file 1 — Supplementary [file ADVS-5-1800714-s001.pdf]

Copyright WILEY-VCH Verlag GmbH & Co. KGaA, 69469 Weinheim, Germany, 2018.

ADVANCED SCIENCE Open Access

Supporting Information

for *Adv. Sci.,* DOI: 10.1002/advs.201800714

Phototunable Biomemory Based on Light-Mediated Charge Trap

Ziyu Lv, Yan Wang, Zhonghui Chen, Long Sun, Junjie Wang, Meng Chen, Zhenting Xu, Qiufan Liao, Li Zhou, Xiaoli Chen, Jieni Li, Kui Zhou, Ye Zhou, Yu-Jia Zeng, Su-Ting Han,* and Vellaisamy A. L. Roy**

Copyright WILEY-VCH Verlag GmbH & Co. KGaA, 69469 Weinheim, Germany, 2016.

Supporting Information

Photo−Tunable Biomemory based on Light-Mediated Charge Trap

Ziyu Lv, Yan Wang, Zhonghui Chen, Long Sun, Junjie Wang, Meng Chen, Zhenting Xu, Qiufan Liao, Li Zhou, Xiaoli Chen, Jieni Li, Kui Zhou, Ye Zhou, Yu-Jia Zeng, Su-Ting Han,* and Vellaisamy A. L. Roy**

Contents:

1. Materials and instruments
2. Device Fabrication
3. The ratio influence of CDs-silk mixed solution on memory properties
4. The concentration influence of CDs-silk mixed solution on memory properties
5. The cell area dependence of ON and OFF current in Ag/CDs-silk/ITO, Al/CDs-silk/ITO and Au/CDs-silk/ITO
6. Distribution of SET voltages of Au/CDs-silk/ITO devices without and with UV light treatment
7. Distribution of SET voltages of Ag/CDs-silk/ITO devices without and with UV light treatment
8. The endurance of Ag/CDs-silk/ITO device over 100 cycles
9. The temperature dependence of currents in HRS and LRS of Al/CDs-silk/ITO device
10. Electrochemical cyclic voltammograms of silk film

1. Materials and instruments

Bombyx mori cocoon was purchased from China National Silk Import and Export Corp. (Beijing, China). Dialysis membrance (MWCO: 3500) was purchased from Spectrum Laboratories Inc. (Shanghai, China). Aqueous solution of carbon dots (10 mg mL^{-1}) were purchased from Janus New-Materials Corp. (Nanjing, China). Sodium carbonate and lithium bromide were purchased from Sigma-Aldrich Corp. (China). Double distilled water (18.2 MΩ cm, MilliQ system) was used in the experiments.

The silk protein solution for device fabrication was prepared as according to a standard protocol. The cut pieces of *Bombyx mori* cocoons were boiled for 60 min in 0.02 M aqueous solution of Na_2CO_3, and then rinsed with double distilled water to remove the glue like sericin and other impurities. The extracted silk fibers were dissolved in 9.3 M lithium bromide (LiBr) solution at 60 ˚C and then dialyzed in double distilled water to remove LiBr. Following centrifuging at 10000 r.p.m. for 15 min to yield silk protein solution, the solution was used for further experiments.

The cross-sectional SEM image of Al/CDs-silk/ITO device was performed with a field emission scanning microscope (Bruker XFlash 6|10). The TEM image of CDs was conducted on a high resolution transmission electron microscope (Tecnai F30). The FTIR-ATR spectrum of silk protein was obtained with a Bruker Vertex 80 V FTIR spectrometer. The circular dichroism spectrum of silk was recorded on a CD spectrometer (J-1500, JASCO). The photoluminescence effect of CDs was investigated via a fluorescence spectrophotometer (RF-5301PC, SHIMADZU). The AFM topographic images were obtained in air in a ScanAsyst mode on a Bruker Dimension Icon AFM (probe: ScanAsyst-AIR). The electrical characteristics of the devices were conducted on a Keysight B1500A parameter analyzer in ambient conditions.

The investigation of charge trapping capacity was using AFM electrical technique. All nanoelectrical measurements were performed in ambient conditions at room temperature on a

Bruker Dimension Icon AFM. SCM-PIT conductive tip (Bruker; Platinum-iridium coating; frequency: 60-100 KHz) was selected to investigate the charge trapping capacity of CDs-silk film on Si substrate. A combination of contact mode and KPFM mode was used. There are two steps for each measurement: two-dimensional charge injection and surface potential measurement. First, in contact mode, at a scan rate of 0.8 Hz, -6 V bias was applied on the conductive tip for the injection of electrons (scanning area: 1 μm × 1 μm; resolution: 256 pixels × 256 pixels). The injection of holes was achieved at a different scanning area by a +6 V tip bias. In a control group, we illuminated the CDs-silk film with a UV light source (λ = 365 nm; intensity: 0.15 mW cm^{-2}) during the whole process of electron and hole injection. Then, the AFM system was switched to an amplitude modulation KPFM mode *in situ* for surface potential measurement. The scanning area and scan rate were 10 μm × 10 μm and 0.8 Hz, respectively. The tip-surface distance for surface potential measurement was 75 nm for all samples (resolution: 256 pixels × 256 pixels).

2. Device Fabrication

Vertical structure device Fabrication. The pattern bottom electrodes were deposited by sputter coating 200 nm thick ITO on to PET substrates through a shadow mask. Then, CDs-silk films with different concentration and/or ratio were spin-coated on the substrate and annealed in ambient conditions at 60 ˚C. Next, the pattern top electrodes were prepared by thermal evaporating Al (Au or Ag) on to the switching films through a shadow mask. Figure S1 displays the optical image of the vertical structure memory device.

Planar structure Fabrication. First, CDs-silk film was spin-coated on a plasma cleaned Si/SiO_2 substrate and annealed in ambient conditions at 60 ˚C. Then, two opposed electrodes (thickness: 30 nm; gap: 20 μm) were prepared by thermal evaporating 30 nm thick Ag on to the CDs-silk film through a shadow mask.

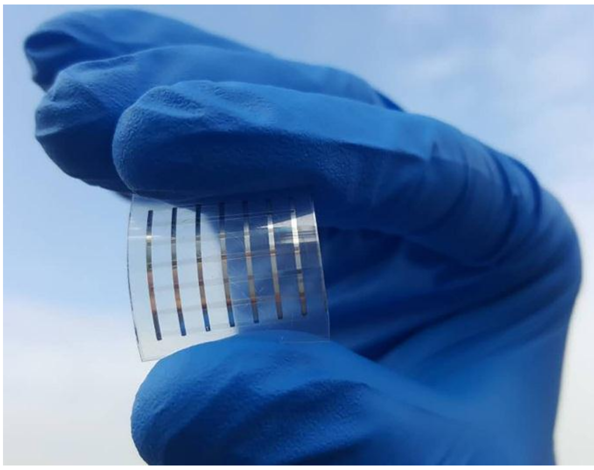

Figure S1. The optical image of the flexible CDs-silk memory devices.

3. The ratio influence of CDs-silk mixed solution on memory properties

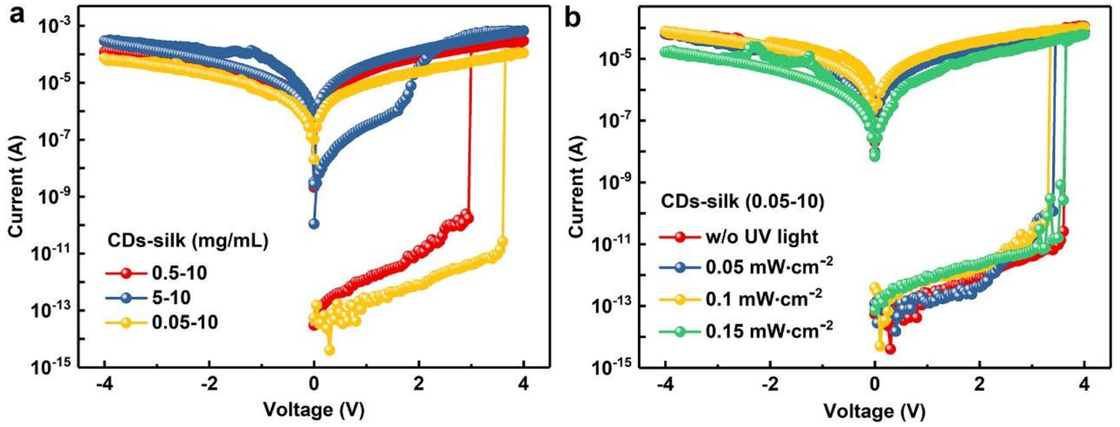

Figure S2. The ratio influence of CDs-silk solution for active layer preparation on Al/CDs-silk/ITO memory properties. (a) I-V characteristics of Al/CDs-silk/ITO devices (compliance current: 1 mA, sweeping rate: 50 mV, CDs-silk solution concentration: 0.5 mg/mL CDs–10 mg mL^{-1} silk (0.5-10), 5 mg mL^{-1} CDs–10 mg mL^{-1} silk (5-10), 0.05 mg mL^{-1} CDs–10 mg mL^{-1} silk (0.05-10)). (b) I–V characteristics of Al/CDs-silk/ITO device (0.05-10) exposed to UV light (λ = 365 nm) with intensity from 0 to 0.15 mW/cm^{-2} (Compliance current: 1 mA, sweeping rate: 50 mV). By comparison, we find that the 0.5-10 group and the 0.05-10 group exhibited a larger ON/OFF current ratio. Whereas, the 5-10 group did not showed a "step-like" increase in the current, which may originate from excessive doping of CDs. In addition, compared with 0.5-10 group (Figure 2c in the main text), the influence of UV illumination on the switching voltage was much weaker in the 0.05-10 group. Hence, for achieving optimum performance, we used the mixed aqueous solution with a concentration of CDs (0.5 mg mL^{-1}) and silk (10 mg mL^{-1}).

4. The concentration influence of CDs-silk mixed solution on memory properties

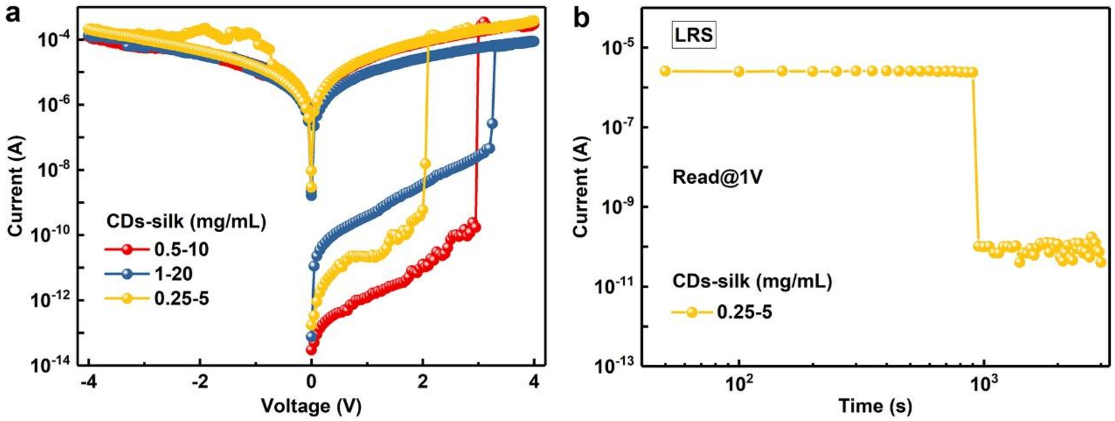

Figure S3. The concentration influence of CDs-silk solution for active layer preparation on Al/CDs-silk/ITO memory properties. (a) I-V characteristics of Al/CDs-silk/ITO devices (compliance current: 1 mA, sweeping rate: 50 mV, CDs-silk solution concentration: 0.5 mg mL^{-1} CDs–10 mg mL^{-1} silk (0.5-10), 1 mg mL^{-1} CDs–20 mg mL^{-1} silk (1-20), 0.25 mg mL^{-1} CDs–5 mg mL^{-1} silk (0.25-5)). (b) The stability of on current of Al/CDs-silk/ITO devices (CDs-silk solution concentration: 0.25 mg mL^{-1} CDs–5 mg mL^{-1} silk). The devices were measured with constant application of 1 V read voltage at 300K. By comparison, we find that the 0.5-10 group and the 0.25-5 group exhibited a larger ON/OFF current ratio. Whereas, the 0.25-5 group with suffered with an instability of LRS state, then retention of the on current is less than 10^3 s. Hence, for achieving optimum performance, we used the mixed aqueous solution with a concentration of CDs (0.5 mg mL^{-1}) and silk (10 mg mL^{-1}).

5. The cell area dependence of ON and OFF current in Ag/CDs-silk/ITO, Al/CDs-silk/ITO and Au/CDs-silk/ITO

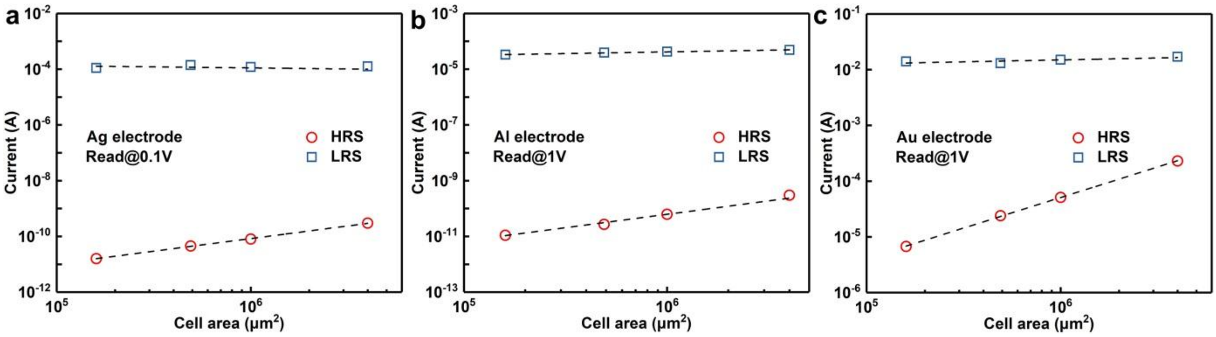

Figure S4. Cell area dependence of ON and OFF current in (a) Ag/CDs-silk/ITO, (b) Al/CDs-silk/ITO and (c) Au/CDs-silk/ITO. The OFF current is proportional to the cell area, as illustrated the Figure below. This variation is due to the same current flowing through the whole cell. While, the ON current is size-independent (device with Ag electrode) or very weak area dependent (device with Al or Au electrode), suggesting that the ON state is mainly attributed to localized conductive channel.

6. Distribution of SET voltages of Au/CDs-silk/ITO devices without and with UV light treatment

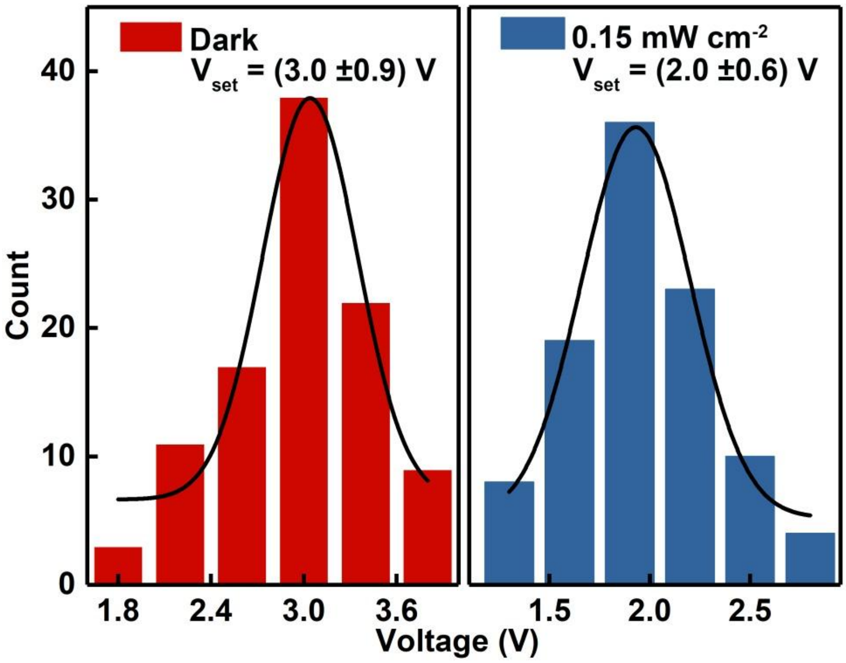

Figure S5. Distribution of SET voltages without (left) and with (right) UV light treatment (100 sample Au/CDs-silk/ITO devices for both statistics histograms). As suggested by the fitting curves, the average values of Vset without and with light irradiation were 3.0±0.9 V and 2.0±0.6 V, respectively. These finding further validated previously observed the UV light irradiation impact on the memory switching voltage.

7. **Distribution of SET voltages of Ag/CDs-silk/ITO devices without and with UV light treatment**

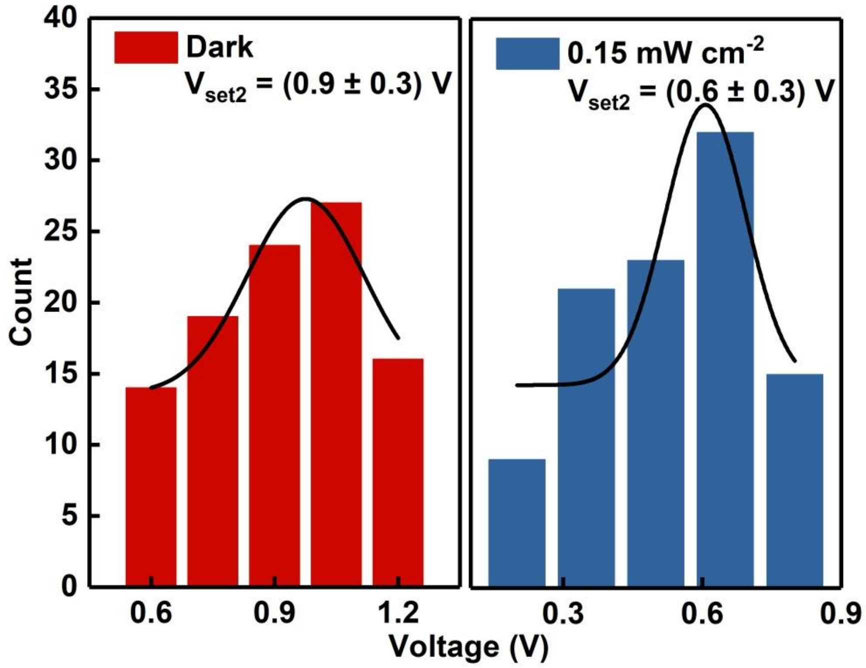

Figure S6. Distribution of SET2 voltages without (left) and with (right) UV light treatment (100 sample Ag/CDs-silk/ITO devices for both statistics histograms). As suggested by the fitting curves, the average values of Vset without and with light irradiation were 0.9±0.3 V and 0.6±0.3 V, respectively. These finding further validated previously observed the UV light irradiation impact on the memory switching voltage.

8. The endurance of Ag/CDs-silk/ITO device over 100 cycles

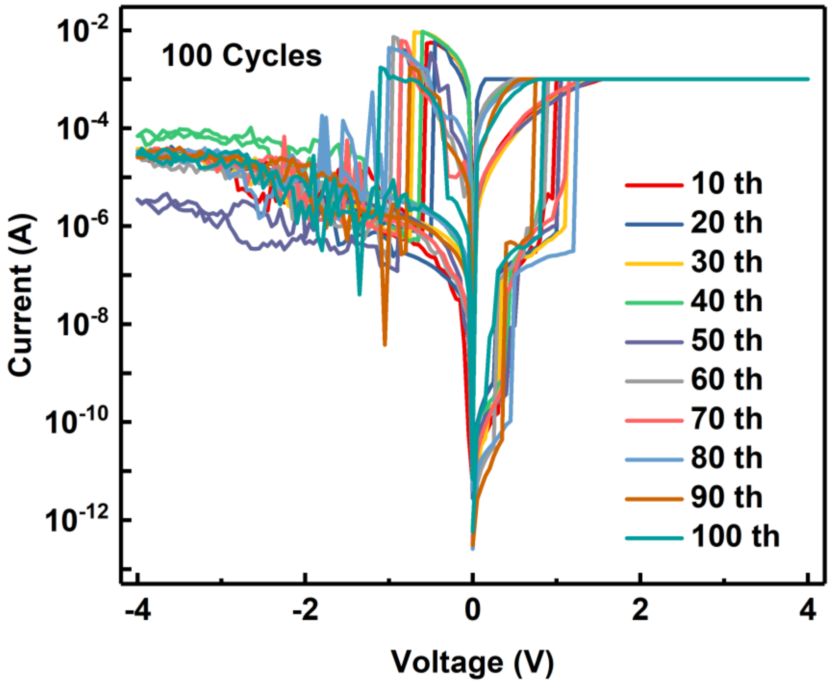

Figure S7. Switching endurance of Ag/CDs-silk/ITO device measured up to 100 cycles, demonstrating the device reliability. Compliance current: 1 mA, sweeping rate: 50 mV. No obvious fluctuation is observed for the device even after 100 SET and RESET sweep cycles.

9. Temperature dependence of currents in HRS and LRS of Al/CDs-silk/ITO device

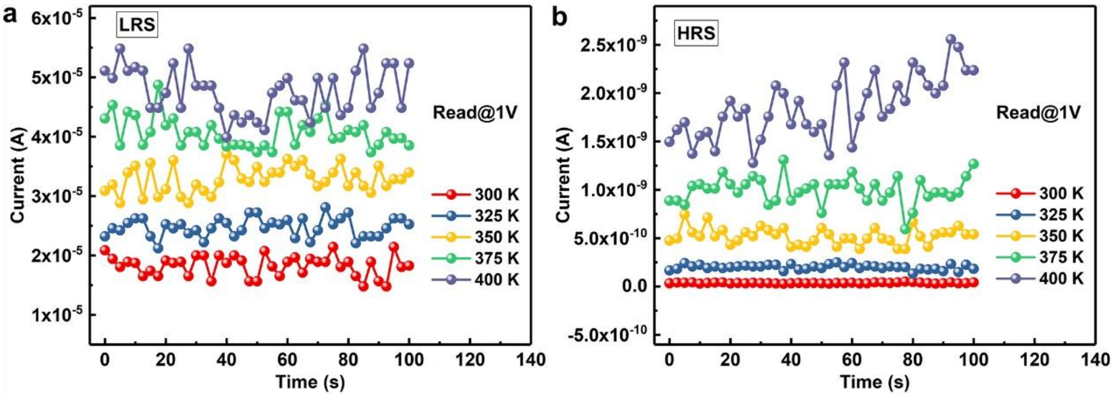

Figure S8. Temperature dependence of currents in LRS (a) and HRS (b) of Al/CDs-silk/ITO device. The device was measured with constant application of 1 V read voltage at 300-400 K. Both on and off current slightly increases with increasing temperature indicating a semiconducting-type or insulating-type conduction, thus excludes the possibility that the formation of metal filament triggers the resistive switching behavior. In particular, strong LRS or HRS current fluctuations were observed at 400 K, which may be caused by protein denaturation.

10. Electrochemical cyclic voltammograms of silk film

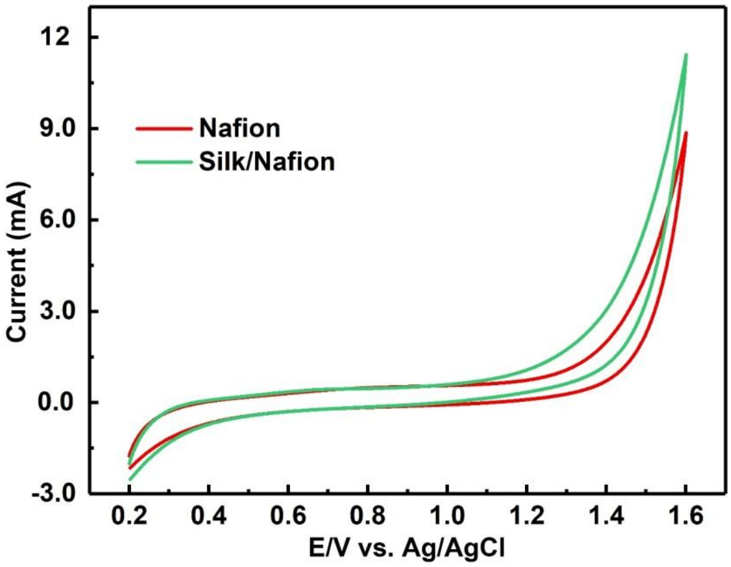

Figure S9. Electrochemical analysis of silk film wrapped with nafion and pure nafion film. Nafion layer was used to protect silk film that may be dissolved by electrolyte during testing. Silk protein film wrapped with nafion coated ITO electrode acted as working electrode. Platinum and Ag/AgCl acted as counter electrode and reference electrode, respectively. 1 M Na_2SO_4 aqueous solution was used as supporting electrolyte. No obvious oxidation/reduction peaks in the voltammograms showed that no obvious oxidation/reduction peaks were detected from voltammograms of both films.
